# Editorial: Editor’s Pick 2021: Highlights in Stem Cell Research

**DOI:** 10.3389/fcell.2022.859472

**Published:** 2022-03-25

**Authors:** Valerie Kouskoff, Atsushi Asakura

**Affiliations:** ^1^ Developmental Hematopoiesis Group, Faculty of Biology, Medicine and Health, The University of Manchester, Manchester, United Kingdom; ^2^ Paul and Sheila Wellstone Muscular Dystrophy Center, Department of Neurology, Stem Cell Institute, University of Minnesota Medical School, Minneapolis, MN, United States

**Keywords:** stem cell, regenerative medicine, embryonic stem cell, development, induced pluriopotent stem cells

Current knowledge of stem cell biology, as well as various cell culture technologies that have been established in recent years, supports the field of developmental biology and regenerative medicine (Ikeya et al., 2021a, b). Stem cells include embryonic stem cells (Martin, 1981; Thomson et al., 1998) with pluripotent differentiation ability retained by fertilized eggs, and induced Pluripotent Stem (iPS) cells (Takahashi et al., 2007; Takahashi and Yamanaka, 2006), which have been developed by cell culture and molecular biological technologies. In addition, mesenchymal stem cells (MSCs) derived from bone marrow are known as multipotent adult stem cells (Gao et al., 2021), capable of differentiating into various mesenchymal cells and have contributed to regenerative medicine as have biological microdevices. Tissue-specific stem/progenitor cells that can be isolated from various adult tissues can contribute to the specific tissue types for physiological tissue maintenance and repair after damage (Prentice, 2019). The use of these stem/progenitor cells has been further enhanced by genetic engineering and embryological strategies (Wang et al., 2021). For example, the use of signal transduction mimicking the developmental stages of tissues to induce specific cell differentiation, the construction of target tissues by organoid culture, a three-dimensional culture method, and the use of exosomes, which play an important role in cell-cell communication, have recently attracted attention. Stem cell biology and regenerative medicine can be systematized by combining these fundamental technologies related to developmental biology and stem cells.

This Editors’ pick research topic aims to highlight a few of the most noteworthy manuscripts published in the Stem Cell section of Frontier in Cell and Development al Biology over 2020 and 2021. The 12 selected manuscripts, highlighted in this topic, were not part of a research topic but have caught our attention and that of the readers by their scope, novelty and quality.

Four primary research manuscripts were chosen to be part of this Editors’ pick. In the first one, Tagliaferri et al. uncovered the molecular mechanism by which retinoic acid induces the transition of embryonic stem cell to a 2-cell like state, implicating DUX and DUXbl1 in this process (Tagliaferri et al.). The second manuscript, by Gao et al. describes the use of patient specific retinal organoids to recapitulate feature of retinitis pigmentosa, establishing the first *in vitro* model system to study the mechanism implicated in the development of this disease (Gao et al.). In the third highlighted manuscript, Chen et al. investigated the role of the Mediator subunit Med23 in adult mouse hippocampal neurogenesis through loss-of-function approach, demonstrating a critical role for Med23 in the regulation of adult brain neurogenesis and function (Chen et al.). In the last primary research manuscript selected for this topic, Chen et al. explored the role of the thyroid hormone T3 on BMP9-induced osteogenesis, demonstrated that both work together to promote osteogenic differentiation via the activation of the AMPK/p38 signalling pathway (Chen et al.).

In addition to these four primary research articles, eight reviews were chosen to complete this collection of noteworthy and recently published manuscripts in the Stem Cell section. These reviews underscore several central themes in this field of research. The therapeutic potential of MSCs was reviewed by Shammaa et al., while Xiong et al. assessed the potential of exosomes from adipose-derived stem cells in tissue regeneration (Xiong et al.). In a related topic, Rees et al. described the latest research on the regenerative properties of intestinal stem cell upon injury (Rees et al.). Two reviews discussed the role and importance of signalling pathways in stem cell maintenance and differentiation. A manuscript by Yang and Jiang describe the role of the signal transducer SMAD2/3 in the control of human embryonic stem cell pluripotency and differentiation (Yang and Jiang), while Rivetti et al. assessed our current knowledge on FGF signalling during mammary gland development, homeostasis and cancer (Rivetti et al.).

On different themes, the physiology of gastric stem cells and potential related-pathologies was reviewed by Xiao and Zhou, while Dumortier et al. summarized the current literature on the role of CFTR mutations on the commitment of induced pluripotent stem cells to bone cells (Dumortier et al.). In the last review selected as part of this topic, Nakamura et al. discussed the role of hypoxia and epigenetics in the regulation of cellular reprogramming (Nakamura et al.).

Selecting manuscripts to include in this Editor’s Pick was an arduous task. This series of twelve articles, that made it to the short list, underscores the remarkable quality of manuscripts recently published in the Stem Cell Research section of Frontiers in Cell and Developmental Biology. This Editor’s Pick also highlights the breadth of stem cell research and regenerative medicine and reveals a field of research moving forward at an incredible pace.
